# Residents' Knowledge regarding Recreational Drug Screening Immunoassays at a Swiss Hospital Group

**DOI:** 10.1155/2024/4400606

**Published:** 2024-06-10

**Authors:** Elias Bekka, Samuel E. Christen, Laura Hermann, Aristomenis K. Exadaktylos, Manuel Haschke, Evangelia Liakoni

**Affiliations:** ^1^Clinical Pharmacology and Toxicology, Department of General Internal Medicine, Inselspital, Bern University Hospital, University of Bern, Bern, Switzerland; ^2^Graduate School for Health Sciences, University of Bern, Bern, Switzerland; ^3^Department of Emergency Medicine, Inselspital, University Hospital Bern, University of Bern, Bern, Switzerland

## Abstract

**Introduction:**

In case of suspected acute recreational drug toxicity, immunoassays are commonly used as diagnostic tools. Although easy to handle, understanding of their limitations is necessary for a correct interpretation of the results. The aim of this project was to investigate residents' knowledge regarding drug screening immunoassays at a Swiss hospital group.

**Methods:**

All residents of a large hospital group in Switzerland were invited by e-mail to participate in an anonymous survey. Following ten multiple choice questions on drug screening tests, the participants were also asked about their demographics, whether they used drug screening tests on a regular basis, and how confident they felt in their ability to interpret test results.

**Results:**

The ten knowledge questions were answered by 110 of the 1026 residents (11%). Among the 108 participants with available demographics, 90% were 25–35 years old, 63% were female, and 70% were at least in their 4^th^ year of residency. The median score of correct answers was 4 out of 10 (range 0–7) and in 50% of the questions, the correct answer was the most frequently selected response. No significant differences in the knowledge scores were found based on the training, confidence level, or the frequency of drug tests used in daily work.

**Conclusion:**

This survey revealed widespread knowledge gaps among residents regarding the interpretation of immunoassay-based drug test results. These findings can be used to implement educational measures on this topic and might provide a basis for targeted information on common pitfalls to be included in laboratory reports.

## 1. Introduction

In patients presenting with suspected acute recreational drug toxicity, drug screening tests can provide a helpful diagnostic tool and are commonly used in emergency departments. Although more specific analytical methods such as liquid or gas chromatography coupled with tandem mass spectrometry [1–3] can detect a large number of substances with high sensitivity and specificity, such methods are more expensive, need specialized personnel, and the results are commonly not available during the acute patient management. Therefore, other analytical methods, i.e., immunoassays, are the tests that are commonly used in most emergency departments. These tests are easy to perform and the results are quickly available. However, these immunoassays use antibodies to qualitatively determine the presence of a specific substance or substance group and have several limitations. For example, especially for the group of amphetamines, false-positive results due to cross-reactivity are very common [4], while a positive result for substances such as tetrahydrocannabinol (THC), whose main metabolite THC-COOH can be detected in urine for weeks after chronic use in some cases, does not necessarily represent acute use [5]. Furthermore, each commercially available immunoassay can detect a specific number of substances or substance groups, and if the suspected substance is not of the test panel, the test will be negative even in case of acute intoxication. Commonly included substances besides THC are cocaine, opiates, and amphetamines, but depending on the assay, other substances or substance groups such as benzodiazepines or phencyclidine (PCP) can also be included. Importantly, novel psychoactive substances such as cathinones and synthetic cannabinoid receptor agonists that have emerged in the last years [6] can typically not be detected with the commonly used drug screen immunoassays.

Due to these aspects, the notion that these immunoassays are easy to use might also be misleading. Although easy to handle (e.g., no special instruments are needed), knowledge of the characteristics of the test and the properties of the suspected substances is necessary for a correct interpretation of the results. Depending on the clinical situation, a wrong interpretation might remain without consequences or go unnoticed (e.g., if the test result does not affect the clinical management), or it could lead to a misdiagnosis and jeopardize patient management (e.g., in the case of a false-negative result) or strain the patient-physician relationship (e.g., in the case of a false-positive result). Prior surveys from other countries among family and primary care physicians [7, 8] and internal medicine residents [9] have demonstrated that a considerable proportion of the participants were not adequately trained regarding the interpretation of such tests thus highlighting the importance of educational measures focussing on this topic.

The aim of this project was to investigate residents' knowledge regarding the interpretation of drug screening immunoassays at the Insel Hospital Group including the University Hospital Bern, a tertiary hospital and one of the biggest healthcare providers in Switzerland, in order to provide a basis for strategies to optimize residents' training regarding drug screening tests.

## 2. Materials and Methods

The survey was conducted electronically using the SurveyMonkey tool which is supported by the University Hospital Bern. The Ethics Committee of the canton Bern reviewed the study and exempted it from approval (Req-2022-00979). Potential participants (all resident physicians employed by the Insel Hospital Group irrespective of discipline) were invited by e-mail to voluntarily and anonymously participate. No power analysis was performed for this descriptive, cross-sectional study, and the final number of included participants depended on the return rate.

The final questionnaire consisted of questions modified from previous similar surveys [7, 9] and questions created by two of the authors based on experiences from clinical practice. Before distribution, the questionnaire was sent to physicians active in the fields of clinical pharmacology and toxicology (*n* = 8), emergency (*n* = 1), internal medicine (*n* = 1), and anesthesiology (*n* = 1) for pilot testing regarding clarity. The final questionnaire was distributed by e-mail with a link to the electronic survey, followed by a reminder e-mail after two weeks. The survey was open for a total of one month (from January 30 to February 28, 2023).

The participants could choose between a German and a French version of the questionnaire. A short description of the drug screening test used at the University Hospital Bern (“Triage® TOX Drug Screen,” manufactured by Quidel Cardiovascular Inc., San Diego, USA), urine immunoassay for amphetamines, methamphetamines, barbiturates, benzodiazepines, cocaine, methadone metabolite (EDDP), opiates, tetrahydrocannabinol (THC), and tricyclic antidepressants [10], which represents a typical panel included in clinically used urine drug tests [11]) was included in the preface. Participants were then asked to answer ten multiple choice questions, which referred to typical urine drug tests such as the one used at our institution, unless otherwise specified. For each question, five possible answers were provided and to continue, one answer had to be chosen. As a result of this requirement, there were no missing data in the survey answers to the knowledge questions. Following the 10 questions on drug test interpretation, a series of general and demographic questions was asked, including participant's sex, age group, current hospital department and position (resident or senior resident), current year of training, whether they use or order drug screening tests on a regular basis, how confident they feel in their ability to interpret the results of urine drug tests (5-point Likert scale, 1 = not at all confident and 5 = very confident), and how many standard urine drug screening tests they already have ordered, performed, or interpreted during their medical training (0, 1–100, or >100).

The primary outcome was the knowledge score, calculated by giving one point for each correct answer (maximum possible score: 10). Numerical data are presented as median and range if not normally distributed and categorical data as number of cases and percentages. Differences between the two groups were explored using the Mann–Whitney test for nonnormally distributed variables and a *p* value of <0.05 was considered statistically significant. The association between sex, training level, confidence, and prior experience with drug tests as independent variables and the primary outcome (knowledge score) as the dependent variable was investigated using multiple regression models. We used both a linear regression model with the primary outcome (the 10-item knowledge score) expressed as a continuous variable and an ordered logistic regression model with the knowledge score expressed as an ordinal variable. A test for internal consistency was performed a posteriori for the 10-item questionnaire using McDonald's omega. Analyses were conducted using the R statistical package (version 4.1.2), R package “psych” and RStudio (2021.09.02). Data visualization was performed with GraphPad Prism version 8.0.1 (GraphPad Software, La Jolla California, CA).

## 3. Results

The questionnaire was sent to 1026 residents working at the Insel Hospital Group. Among these, 110 (10.7%) completed the questionnaire on drug test knowledge and 108 also provided data on training and demographics (Table 1).

Thirty-four participants (31.5%) were from the General Internal Medicine department, followed by residents in paediatrics (*n* = 12, 11.1%), neurology (*n* = 8, 7.4%), and anesthesiology (*n* = 7, 6.5%). The departments of angiology, diabetology and endocrinology, intensive care medicine, emergency medicine (adults), orthopaedic and traumatology, and pneumology had four participants each, while departments with fewer participants were gynecology (three participants, 2.8%), surgery, neurosurgery, gastroenterology, emergency medicine (children), visceral surgery, and “other” (two participants (1.9%) each), otorhinolaryngology, hematology, infectiology, oral and maxillofacial surgery, nephrology, osteoporosis, and radiology (one participant (0.9%) each).

**Figure 1:**
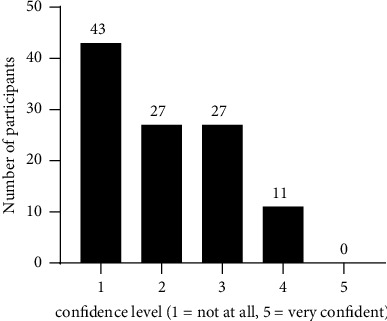
Participants' (*N* = 108) perception regarding their ability to interpret urine drug tests.

**Figure 2:**
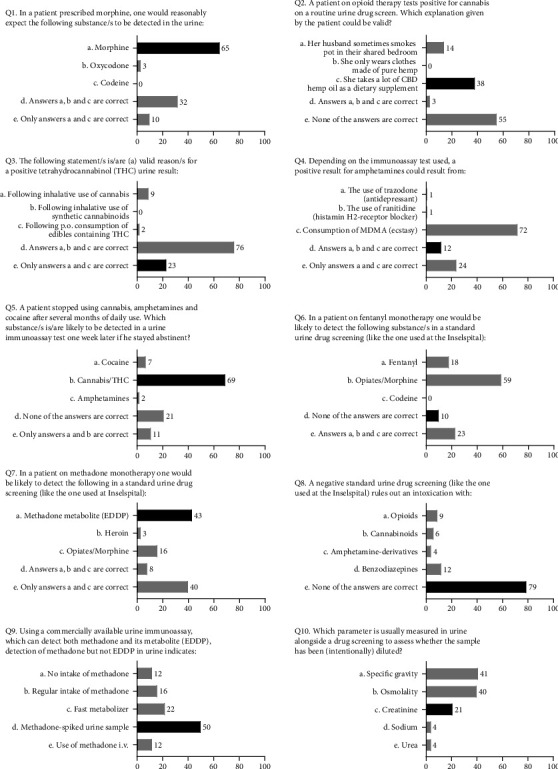
Participants' (*N* = 110) answers to the knowledge questions (correct answers shown in black).

**Figure 3:**
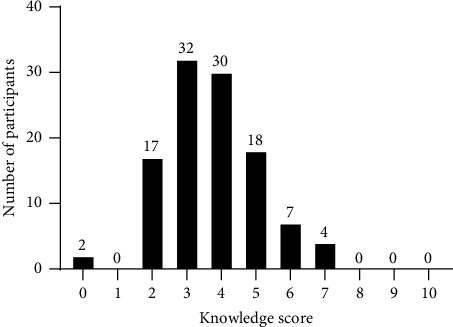
Distribution of knowledge score based on the number of correct answers (*N* = 110).

The participants' own perception regarding their ability to interpret the results of urine drug tests is shown in Figure 1; the results of the ten multiple choice knowledge questions are presented in Figure 2. The median of correct answers to the ten knowledge questions was 4 (range: 0–7), and the score distribution is shown in Figure 3. The McDonald's omega (total) was 0.39, indicating a low internal consistency.

On bivariate analysis, there were no significant differences in the knowledge score between participants with 1–3 and ≥4 years of residency (*p*=0.59), confidence level 1-2 and ≥3 (*p*=0.72), or based on the use or order of drug tests on a regular basis (*p*=0.51). There were also no significant differences in the knowledge score when stratifying the analysis by sex or when accounting for sex, training, confidence, and drug test use in multivariable models.

## 4. Discussion

In this anonymous, electronic survey, resident physicians at the Insel Hospital Group most frequently selected the correct answer in 5 of the ten questions. More than one-third of the participants did not feel confident at all in their ability to interpret drug screening results and the majority of the participants had a knowledge score of <5/10 points. These results are broadly in line with prior research on drug knowledge in general practitioners, internal medicine residents, psychiatry residents and fellows, as well as pediatricians [7–9, 12]. As these studies were conducted several years ago and had a more narrow focus (e.g., on specific drugs such as opioids or specific medical disciplines), the findings of this survey expand prior observations and suggest that the poor proficiency in drug test interpretation remains an ongoing issue for several substances included in a typical urine drug test panel and is generalizable to the setting of a hospital group (including a large tertiary care university center as well as community hospitals), encompassing various disciplines and levels of training.

Similar to the studies of Reisfeld et al. [7], Starrels et al. [9], and Suzuki et al. [12], knowledge was not associated with prior experience in drug test use. However, in contrast to Starrels et al. who saw significant differences in knowledge between interns and more experienced residents and divergent correlations between knowledge and confidence when stratifying according to sex (negative correlation in male participants and positive correlation in female participants), neither sex nor training level were associated with the knowledge score in our study. The lack of effect of both professional experience and prior drug test use on knowledge likely reflects in part the insufficient emphasis placed on drug test interpretation during medical education and training.

One aspect that seems to cause confusion is the ability of the usual immunoassays to detect and differentiate between various opiates (naturally occurring substances such as morphine) and synthetic opioids, such as methadone and fentanyl. When using an immunoassay drug test that can detect “opiates” as a substance group, a positive result is expected after morphine use, but not following the use of synthetic substances such as oxycodone or fentanyl [9, 12]. Immunoassay tests for synthetic opioids such as methadone and its metabolite EDDP are available, but these results are provided separately from the “opiates” group, which would be expected to be negative in the case of the use of methadone only. Immunoassays for both methadone and EDDP are commonly used in the context of checking for compliance with substitution therapy. A positive result for methadone and EDDP indicates therapy adherence, a negative result for both would represent no consumption, a positive result for EDDP only indicates fast metabolism (genetically or due to interaction with other substances), and a positive result for methadone only indicates sample adulteration by the addition of methadone directly into the urine [13]. When using immunoassays for detecting opiates, it is important to consider that both heroin and codeine are metabolized to morphine [13]. Depending on the time since use, this could lead to a positive result for “opiates” when using these tests, due to the detection of morphine and not of heroin or codeine directly. Since heroin is first metabolized to 6-monoacetylmorphine (6-MAM), some immunoassays available on the market can additionally detect 6-MAM as a specific marker for heroin use, but due to the short detection time window for 6-MAM (elimination half-life: 6–25 minutes), morphine might be the only detectable substance following heroin use [13]. Codeine is metabolized to morphine by cytochrome P450 2D6 (CYP2D6). The rate of this step is, among others, influenced by CYP2D6 genetic polymorphisms, leading to some people being “ultrarapid” and other people being “poor” metabolizers [13, 14]. Besides codeine-containing drugs, poppy seeds also contain traces of opiates and can thus lead to a positive result [13, 15].

Similar to naturally occurring opiates vs. synthetic opioids, structural differences are also relevant for the detection of THC metabolites vs. synthetic cannabinoids. In contrast to the naturally occurring psychoactive cannabinoid THC, synthetic cannabinoids (or more accurately described as synthetic cannabinoid receptor agonists) are synthetic substances that also act as agonists at the cannabinoid receptors but are not detected in most immunoassays, due to their markedly different structure compared to THC [16, 17]. This aspect seemed to be unknown to most of the participants in our study. Conversely, while pure formulations of the naturally occurring cannabinoid cannabidiol (CBD) usually do not show up on drug screening tests [18], oral ingestion of hemp products can lead to a positive cannabis test result [19], due to varying THC content reflecting jurisdiction-dependent legal limits (usually ranging from 0.2 to 1% THC content for hemp) [20]. THC metabolites can be detected for days or weeks after regular cannabis consumption [13, 21], without representing recent use or acute intoxication. This seemed to be better known among participants compared to the aspects mentioned above. On the other hand, except in the case of massive exposure in unventilated areas, second-hand smoke is very unlikely to produce false-positive results if a cutoff of 50 ng/ml THC-COOH is used [22], as is the case for the immunoassay used at our institution.

Knowledge about cross-reactivities in drug tests was not widespread based on the results of the survey, even though false-positive results due to interacting substances are well described, mainly for the amphetamine component of drug screening immunoassays [23]. The antidepressants trazodone and bupropion, the H2-antihistamine ranitidine, and stimulants such as ephedrine or methylphenidate are among the substances that can cross-react with amphetamines in immunoassays [24]. Information on the product-specific cross-reactivities, as well as the cutoff values for the specific substances, is available in the package insert of the commercially available immunoassays (commonly also available online). Confirmatory testing with more specific methods (e.g., chromatography coupled with mass spectrometry) should be considered in unclear situations, especially when confronted with a positive amphetamine immunoassay result. In contrast, the test for the cocaine metabolite benzoylecgonine displays a more favorable specificity for recent cocaine exposure, although intriguingly false-positive results have been reported in patients undergoing evaluation for organ transplantation [25].

Most survey participants thought that either specific gravity or osmolality rather than creatinine was the most commonly used marker to detect purposefully diluted samples, a scenario which might be encountered when testing for drugs of abuse. If the urine creatinine is below 20 mg/dL, the sample is usually considered too diluted to adequately reflect a patient's drug exposure and hence does not allow reliable interpretation [26]. Quantification of creatinine concentration is usually the preferred method to screen for dilution in clinical practice due to the ease of measurement [27]. In guidelines for workplace drug testing, specific gravity is recommended as a secondary measurement to confirm dilution if urine creatinine is less than 20 mg/dL [28]. While a low urine osmolality can also indicate dilution, it is rarely used to assess the validity of drug screening tests because the method is time consuming [27].  Table 2 summarizes some important aspects to consider when interpreting drug test immunoassays.

To remedy this lack of knowledge and improve urine drug test interpretation by physicians, several options could be considered. This study's findings of knowledge gaps irrespective of training status may indicate that this topic should be emphasized more prominently at multiple levels of medical training and continuous education, e.g., in pregraduate medical education, during postgraduate training, and as information provided to the practicing physician. In general, education on laboratory medicine topics is not preeminently featured in medical school curricula [29]. Its teaching is often limited to theoretical aspects and eschews intricacies that might be encountered clinically later on. Discussions on the strengths and limitations of laboratory-based diagnostic methods such as drug tests should be considered in future attempts to standardize medical education in laboratory sciences and analytical chemistry [30]. During residency, emphasis could be placed on challenges in drug test interpretation that are likely to be of high clinical relevance. Fields making frequent use of these tests (e.g., emergency and addiction medicine) should consider integrating education measures on this topic as integral parts of their curricula to be explored on grand rounds or as case-based learning. Most useful would probably be to provide the information just in time (i.e., when and how it is needed) to the ordering physician. This can be achieved by including short comments on the main pitfalls in drug test interpretation (Table 2) next to the test results and by adding a reference to the laboratory guide for a more in-depth discussion of general and specific test-related limitations. Finally, encouraging clinicians to contact the laboratory to assist in the interpretation of unclear cases by explicitly mentioning this possibility in the report might lead to quality improvements [31].

This study had several limitations. The relatively low return rate (10.7% of the invited participants) compared to previous studies (40–75%, corresponding to 60–359 participants [7–9]) and associated small sample sizes limited statistical power to detect significant differences in drug test knowledge in groups of interest. A test of internal consistency performed a posteriori showed poor reliability, which is likely attributable to the fact that the test items measure very different aspects of the drug test interpretation proficiency, assessing knowledge of different substances and test characteristics and using questions of varying levels of difficulty. However, our aim was not to design a standardized testing instrument but to increase awareness among residents about common pitfalls of drug test interpretation in clinical practice. This resulted in a small number of markedly different questions, which negatively affected reliability in retrospect. As participation in this study was voluntary, a significant sampling bias is likely, e.g., participation of mostly motivated residents, knowledge of nonparticipants could thus have been even lower. Reporting of prior drug test use was based on self-assessment which is subject to recall bias. It cannot be excluded that some participants consulted the internet for the correct answer. The survey questionnaire was newly developed for this study and was not a previously validated assessment tool. While it was designed based on prior research and examples taken from clinical practice and also was pilot-tested by experienced physicians, it is unclear whether it represents an optimal instrument for the evaluation of medical trainees. For instance, some questions were very specific and probably very difficult for most physicians who do not regularly use drug tests in their daily routine. This could have also contributed to the very low return rate.

In conclusion, this project identified widespread knowledge gaps regarding drug test immunoassays among medical residents of a hospital group, across several disciplines and levels of training. These findings can guide the implementation of specific educational and quality improvement measures such as teaching sessions and targeted information in laboratory reports. The correct interpretation of a drug test immunoassay may be challenging in clinical practice since having an understanding of the specific immunoassay used, its limitations (e.g., cutoff values and cross-reactivities), and the pharmacology of the substances of interest (e.g., metabolic pathways and detection windows) is necessary to avoid misinterpretations that might endanger patient management or negatively affect the patient-physician relationship. In unclear cases, consultation with specialists (e.g., the hospital's laboratory or specialized toxicology or forensics units) is recommended.

## Figures and Tables

**Table 1:** Participants' demographics and training characteristics (*N* = 108).

	*n* (%)
*Sex*	
Female	68 (63.0)
Male	38 (35.2)
Diverse/other nonbinary/no answer	2 (1.9)

*Age*	
<25 years	1 (0.9)
25–30 years	39 (36.1)
31–35 years	58 (53.7)
>35 years	10 (9.3)

*Year of residency*	
1 year	13 (12.0)
2 year	4 (3.7)
3 year	15 (13.9)
4 year	20 (18.5)
5 year	23 (21.3)
6 year	15 (13.9)
>6 year	18 (16.7)

*Position*	
Resident	97 (89.8)
Senior resident	11 (10.2)

*Use or order of drug screening tests on a regular basis*	
Yes	20 (18.5)
No	88 (81.5)

*Number of standard urine drug screening tests ordered, conducted, or evaluated*	
0	27 (25.0)
1–100	80 (74.1)
>100	1 (0.9)

**Table 2:** Limitations and pitfalls to consider when interpreting drug test immunoassay results (selection) [9, 12, 13, 17, 18, 22, 25–29].

(i) Cross-reactivity with other compounds can cause false-positive results (common issue within the amphetamine group and very unlikely in the case of cocaine metabolite testing).
(ii) Possible reasons for a false-negative result include the test's inability to identify a specific compound (e.g., novel psychoactive substances), concentrations below the assay's cutoff, and a short detection window of the substance of interest (e.g., GHB and 6-MAM).
(iii) Immunoassays for “opiates” typically do not detect synthetic opioids (e.g., oxycodone and fentanyl); similarly, immunoassays for THC metabolites do not adequately detect synthetic cannabinoids and Z-drugs (e.g., zolpidem) give a negative result for “benzodiazepines”.
(iv) 6-MAM is a specific marker for heroin use that can be detected with some immunoassays shortly after use; otherwise, a positive result for “opiates” does not allow differentiation between heroin, codeine, and morphine use.
(v) Following regular/chronic cannabis use, THC metabolites can be detected in urine for several weeks without necessarily representing acute intoxication.
(vi) Some commonly abused substances such as ketamine, GHB, LSD, and novel psychoactive substances (e.g., synthetic cathinones and synthetic cannabinoids) are not commonly included in most drug test immunoassays currently in clinical use, which might lead to a negative result despite acute intoxication.
(vii) Additional analytical methods (e.g., chromatography coupled with mass spectrometry) can provide more information in unclear cases and are thus recommended especially in cases with potential legal consequences.
(viii) Validity of urine samples should be assessed by measuring creatinine concentrations to detect dilution; when urine creatinine is below 20 mg/dL, interpretation is not recommended.

6-MAM, 6-monoacetylmorphine; GHB, *γ*-hydroxybutyrate; LSD, lysergic acid diethylamide; THC, tetrahydrocannabinol.

## Data Availability

The data used to support the findings of this study are included within the article.
